# A Coordinated Analysis of the Associations Among Personality Traits, Cognitive Decline, and Dementia

**DOI:** 10.1093/geroni/igab046.035

**Published:** 2021-12-17

**Authors:** Eileen Graham, Kathryn Jackson, Bryan James, Emily Willroth, Daniel Mroczek

**Affiliations:** 1 Northwestern University, Chicago, Illinois, United States; 2 Rush University, Chicago, Illinois, United States

## Abstract

There are considerable individual differences in the rates of cognitive decline across later adulthood. Personality traits are among the factors that may account for some of these differences. The current project investigated whether personality traits were associated with trajectories of cognitive decline, and whether the associations were different before and after dementia diagnosis. The data were analyzed using linear mixed effect regression models. Across study aims was a focus on replicability and generalizability. Each question was address in four independent longitudinal studies (EAS, MAP, ROS, SATSA), and then meta-analyzed using random effects meta-analysis, providing estimates of heterogeneity. As expected, we detected evidence for cognitive decline in all four samples. Results also indicated that neuroticism and openness were associated with total cognitive function. and openness was associated with decline post dementia diagnosis.

